# Bioactive–hybrid–zirconia implant surface for enhancing osseointegration: an in vivo study

**DOI:** 10.1186/s40729-018-0129-3

**Published:** 2018-06-14

**Authors:** Dawlat Mostafa, Moustafa Aboushelib

**Affiliations:** Dental Biomaterials, Faculty of Dentistry, Alexandria University, Champolion St., Azarita, Alexandria, Egypt

**Keywords:** Zirconia implants, Selective etching, Hybrid ceramic surface, SEM

## Abstract

**Background:**

Zirconia is characterized by a hard, dense, and chemically inert surface which requires additional surface treatments in order to enhance osseointegration. The proposed hypothesis of the study was that combination of a nano-porous surface infiltrated with a bioactive material may enhance osseointegration of zirconia implants.

**Methods:**

Custom-made zirconia implants (3.7 mm × 8 mm) were designed, milled, and sintered according to manufacturer recommendations. All implants received selective infiltration etching (SIE) technique to produce a nano-porous surface. Surface porosities were either filled with nano-hydroxy apatite particle- or platelet-rich plasma while uncoated surface served as a control (*n* = 12, *α* = 0.05). New surface properties were characterized with mercury porosimetry, XRD analysis, SEM, and EDX analysis. Implants were inserted in femur head of rabbits, and histomorphometric analysis was conducted after healing time to evaluate bone–implant contact percentage (BIC%).

**Results:**

Selective infiltration etching produced a nano-porous surface with interconnected surface porosities. Mercury porosimetry revealed a significant reduction in total porosity percent after application of the two coating materials. XRD patterns detected hexagonal crystal structure of HA superimposed on the tetragonal crystal phase of zirconia. Histomorphometric analysis indicated a significantly higher (*F* = 14.6, *P* < 0.001) BIC% around HA–bioactive–hybrid surface (79.8 ± 3%) and PRP-coated surface (71 ± 6 %) compared to the control (49 ± 8%).

**Conclusions:**

Bioactive–hybrid–zirconia implant surface enhanced osseointegration of zirconia implants.

## Background

Dental implants became one of the most reliable techniques used to restore missing teeth [1, 2]. Material composition and surface topography play a fundamental role in osseointegration [3]. Therefore, various chemical and physical surface modifications have been developed to improve osseous healing around the inserted implants. Two main approaches have been suggested to improve surface properties of dental implants either by optimizing its micro-roughness or through applying bioactive coatings [4–6].

Hydroxyapatite (HA) is the most widely used bioactive-ceramic material in the field of bone regeneration and augmentation because of its unique bioactivity and stability [7]. Hydroxyapatite promotes growth of bone tissue directly on its surface; hence, HA coatings have been applied to implant fixtures to produce a bioactive surface that stimulates faster bone formation and reduced healing time [8–10]. HA promotes cell attachment and proliferation of a variety of cells including fibroblasts, osteoblasts, and periodontal ligament cells [7].

Platelet-rich plasma (PRP) growth factors loaded onto titanium implant surface were tested in animal models as potential agents to enhance osseointegration. PRP protein coat has two important properties that contribute to optimizing and accelerating the osseointegration process: the osteo-conductive properties attributed to fibrin and the recognized osteo-inductive activities of the growth factors, thereby creating a new dynamic implant surface [11].

All-ceramic dental implants gained lots of attention offering a solution to the potential immunologic and possible esthetic compromises observed with titanium implants [12, 13]. The superior mechanical properties of zirconia made it a material of choice for fabrication of dental implants [14]. Zirconia has an exceptional biocompatibility, chemical stability, and high toughness compared to other metallic materials [15, 16]. Zirconia has an opaque whitish color which prevents grayish discoloration observed with thin gingival biotypes [17–20]. Moreover, the inflammatory response and bone resorption induced by released ceramic particles are much less compared to those induced by titanium particles [21, 22]. On the other hand, zirconia is characterized by a hard, dense, and chemically inert surface that does not react readily to etching even by aggressive chemical agents [23]. Poor surface properties resulted in adhesion problems and caused deboning of coating materials [24].

Different surface modifications of zirconia were tested to increase its surface roughness, wetting capacity, and surface energy [25]. Such approaches mainly include topographical modifications via milling, particle abrasion, hot acid etching [25, 26], or laser micro-etching [27, 28]. Improved surface properties enhanced the performance of zirconia implants [29].

In 2007, Aboushelib and Feilzer introduced a surface treatment method known as selective infiltration etching (SIE) technique that uses the principles of heat-induced maturation and grain boundary diffusion to transform the relatively dense and smooth (non-bonding) surface of zirconia into a highly retentive nano-porous surface which greatly enhanced wetting and bonding capacity to zirconia [24]. This principle could also be used to increase retention of bioactive coatings on the surface of selective infiltration etching zirconia implants where the created nano-pores could be filled with the desired coating material creating a novel bioactive–hybrid ceramic surface without the risk of delamination of the coated material. The only consideration is that the filling particles should be smaller in diameter than the average pore diameter to insure proper pore filling.

For a bioactive coat to achieve its intended functions successfully, several factors must be considered; implant surface treatment (either mechanically or chemically), the thickness of the coated film (thicker films have higher tendency to delaminate), and the properties of the coated film (chemical structure, crystal structure, and surface topography) [30]. The aim of this study was to evaluate the influence of novel bioactive–hybrid–zirconia implant surfaces on osseointegration in a rabbit model. The proposed hypothesis was that the bioactive–hybrid surfaces would enhance osseointegration in the tested animal model.

## Methods

### Preparation of zirconia implants

CAD/CAM zirconia milling blocks (NobelBiocare, Göteborg, Sweden) were used for preparation of zirconia implants (cylinders 3.7 mm × 8 mm). The milled implants were sintered according to manufacturer recommendations (1350 °C for 6 h) [24]. To produce a nano-porous surface, all specimens were subjected to selective infiltration etching (SIE) technique to modify their surface topography through the creation of a nano-porous zirconia surface that extends few microns deep below the surface. Further details are mentioned elsewhere [31]. The prepared implants were divided into two groups (*n* = 12), according to the coating used to fill surface porosities, while uncoated surface served as a control.

### Characterization of zirconia surface

Several surface characterization techniques were implemented to study the proposed hypothesis. Mercury porosimetry was performed for testing the surface nano-porosity and its relevant parameters including the total porosity percent and the average pore diameter in nanometers. Poresizer (Porosimeter, Micromeritics 9320, USA) was used for testing the nano-porosity created on the surface covering pore diameter range from approximately 0.006 to 360 μm. Atomic force microscopy (AFM) was used to confirm surface topography on the nano-scale. High-resolution X-ray diffraction analysis (XRD) for thin coats (PANalytical, X Pert PRO) with Cu target (*λ* = 1.54°A), 45 kV, 40 mA, and 2*Ɵ* (10°–80°) was performed to investigate the crystallographic structure of the implants. Zirconia implants were gold sputter-coated (fine coat, JEOL JFC-1100E, Japan) before scanning electron microscopic examination (JEOL, JSM, 5410, Japan) at an accelerating voltage of 25 kV. Energy dispersive X-ray analysis (EDX) was used (INCA Penta FETX3, OXFORD Instruments, Model 6583, England) to study the elemental composition of the specimens.

### Preparation of HA coating material

Natural hydroxyapatite was extracted from femoral bones of line V Spain white rabbits (the animal models of the experimental study) through two stages: the first stage was the deproteinization process that was conducted to eliminate all organic and protein components of bone, followed by a heat treatment (calcination) process of the inorganic bone salts for elimination of all phases other than HA [32]. EDX and XRD analysis were accomplished to characterize the extracted natural HA for its chemical and crystal phase purity. The prepared powder was firstly ground to reduce its particle size to the sub-micron scale using ultrasonic vibration (ESPE.CAPMIX 410630, W-Germany) with a ceramic ball. Afterwards, high-energy ball milling (8000 M Mixer/Mill, SPEX Sample Prep, USA) was used for grinding of the produced micro-particles to reduce their size to the nano-scale (100 nm). The produced nano-particles were collected using high-speed centrifugal unit.

A hydroxyapatite suspension was prepared by weighing 0.2 g of the milled nano-particles using a calibrated digital balance (OHAUS, CT 1200-S, USA) and adding them to 20 ml absolute ethyl alcohol, followed by shaking the suspension in ultrasonic shaker for 5 min to achieve even distribution at room temperature. Immersion coating was performed by completely immersing the prepared implants in the suspension for 3 min, followed by heat drying at 150 °C for 10 min (Nuova II, Sybron/Thermolyne, Spain). This immersion cycle was repeated three times. Finally, all specimens were heated at 850 °C for 3 h to fuse the particles with the surface of zirconia.

### Preparation of platelet-rich plasma coating material

This study was approved by the ethics committee of Science and Technology Development Fund (STDF-389, the academy of scientific research) regarding using animals in research studies. Autologous platelet-rich plasma (PRP) was freshly prepared from each animal independently just before insertion of zirconia implants into the rabbit femurs. Protocol of blood collection from rabbits was approved by the ethics committee of Alexandria University for studies involving animal models [33]. The collected blood was centrifuged at 4000 rpm for 8 min at room temperature. The centrifuged blood was separated into platelet-poor plasma (PPP), PRP (buffy coat), and the more dense red blood cells. The PRP-coating solution was prepared by collecting the buffy coat in another sterile graduated tube, and 0.5 ml of 10% *w*/*v* calcium chloride was added to each 0.1 ml of separated PRP [34]. Each zirconia implant was completely immersed into the prepared PRP solution for 10 min at room temperature immediately before its insertion into its surgically prepared socket that was completely filled with the prepared PRP solution.

### Surgical phase

Twenty-four male line V Spain white rabbits were obtained from the Poultry Research Center, Faculty of Agriculture, Alexandria University (6 months old and 3 kg), in good health. The rabbits were kept at the animal house and provided soft diet enriched with vitamins (General Pharma Group, Egypt) and selenium (Mchandes Pharma Veterinary, Egypt) which were added to the drinking water with dose of 1 ml/l for about 2 weeks before surgery. All surgical procedures were performed under general anesthesia and aseptic conditions; the same surgeon completed all surgical procedures. Animals were randomly divided into two groups (*n* = 12), in each group; each animal received one hybrid–zirconia implant surface coated with either HA or PRP in the right femur head while the left side received uncoated zirconia implant used as a control.

Before surgery, surgical sites were shaved to expose the skin that was coated with antiseptic iodine-based solution. Rabbits were anesthetized with intramuscular injection of ketamine in combination with xylazine at a dose of 35 and 5 mg/kg of body weight respectively. Surgical flap was made and reflected to expose the distal head of the femur; then, sequential drilling of implant socket was carried out under sufficient cooling at room temperature and with an absolute minimum amount of trauma; and afterwards, the implant was inserted into its created socket. Finally, the surgical flap was repositioned and sutured. Postoperative intramuscular injection of broad-spectrum antibiotic (Cefotax 250 mg; Egyptian INT; Pharmaceutical Industrial Co., Egypt) and analgesic (Voltaren 75 mg/3 ml; Novartis Pharma S.A.E. Egypt) were administrated daily for 10 days to avoid any infection and to relief pain. Rabbits were monitored daily for weight gain and cage behavior. The wounds were allowed to heal for 6 weeks before sacrifice by injection of an over dose of intravenous anesthetic agent [35, 36].

### Histomorphometric analysis

Six weeks after insertion of the implants, bone blocks were collected, subjected to fixation and dehydration, imbedded in transparent methyl methacrylate monomer, and finally sectioned in a precision cutting machine using a diamond-coated disc producing 150-μm-thick sections. Sections were polished using silicon carbide and stained using Stevenel’s Blue and van Gieson picro-fuchsin. Histomorphometric analysis and determination of bone-to-implant contact percentage (BIC%) was performed on the mid section of each implant using digital images obtained from a stereo stereomicroscope (Olympus imaging digital camera, model E.330 DC 7.4 V, Japan). The images were then analyzed using computer software program (Olypus. Cell ˆA). Mature bone stained red in contact with implant diameter was measure as a percentage of the entire implant diameter to calculate bone implant contact percent of each test group. Data were fed to a statistical software (SPSS 14.0, SPSS Inc). One-way analysis of variance (ANOVA) and Bonferroni post hoc tests were used to analyze the data based on power analysis test [37, 38].

## Results

**Fig. 1:**
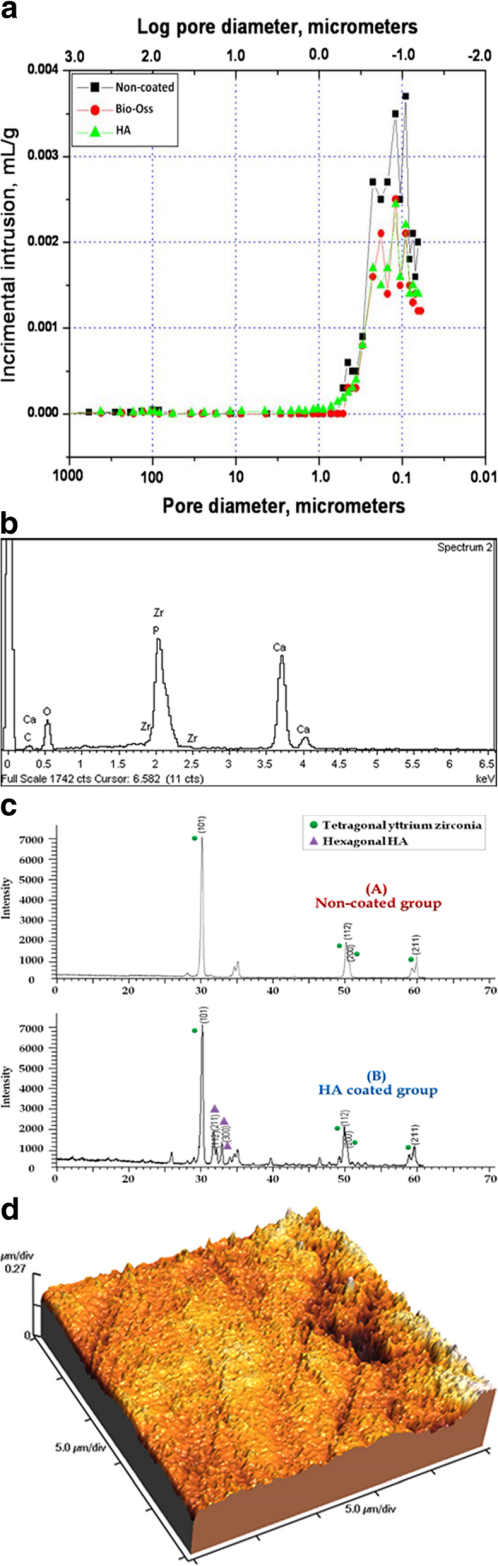
**a** Mercury porosimetry and the average pore diameter of the prepared implants. **b** EDX analysis of hybrid–zirconia surface showing peaks of zirconia, calcium, and phosphate. Ca/P ratio is 1.67. **c** XRD peaks of uncoated and bioactive implants showing characteristic peaks specific for tetragonal yttrium zirconium oxide crystal system represented by (101), (112), (200), and (211) and hybrid implants showing characteristic peaks specific for hexagonal HA crystal system. **d** Atomic force microscope of selective infiltration etching zirconia surface demonstrating subsurface porosities

**Fig. 2:**
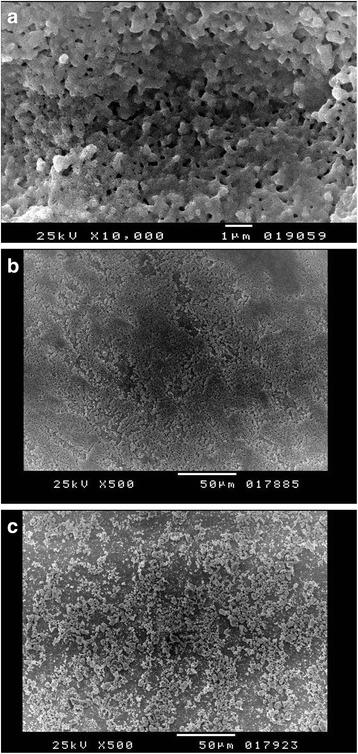
**a** SEM image, ×10,000, demonstrating the characteristic porous surface of selective infiltration etching surface of zirconia. **b** SEM image, ×500, demonstrating deposition of PRP coat and complete filling of the porous surface. **c** SEM image, ×500, demonstrating filling of the porous surface with particles of HA

**Fig. 3:**
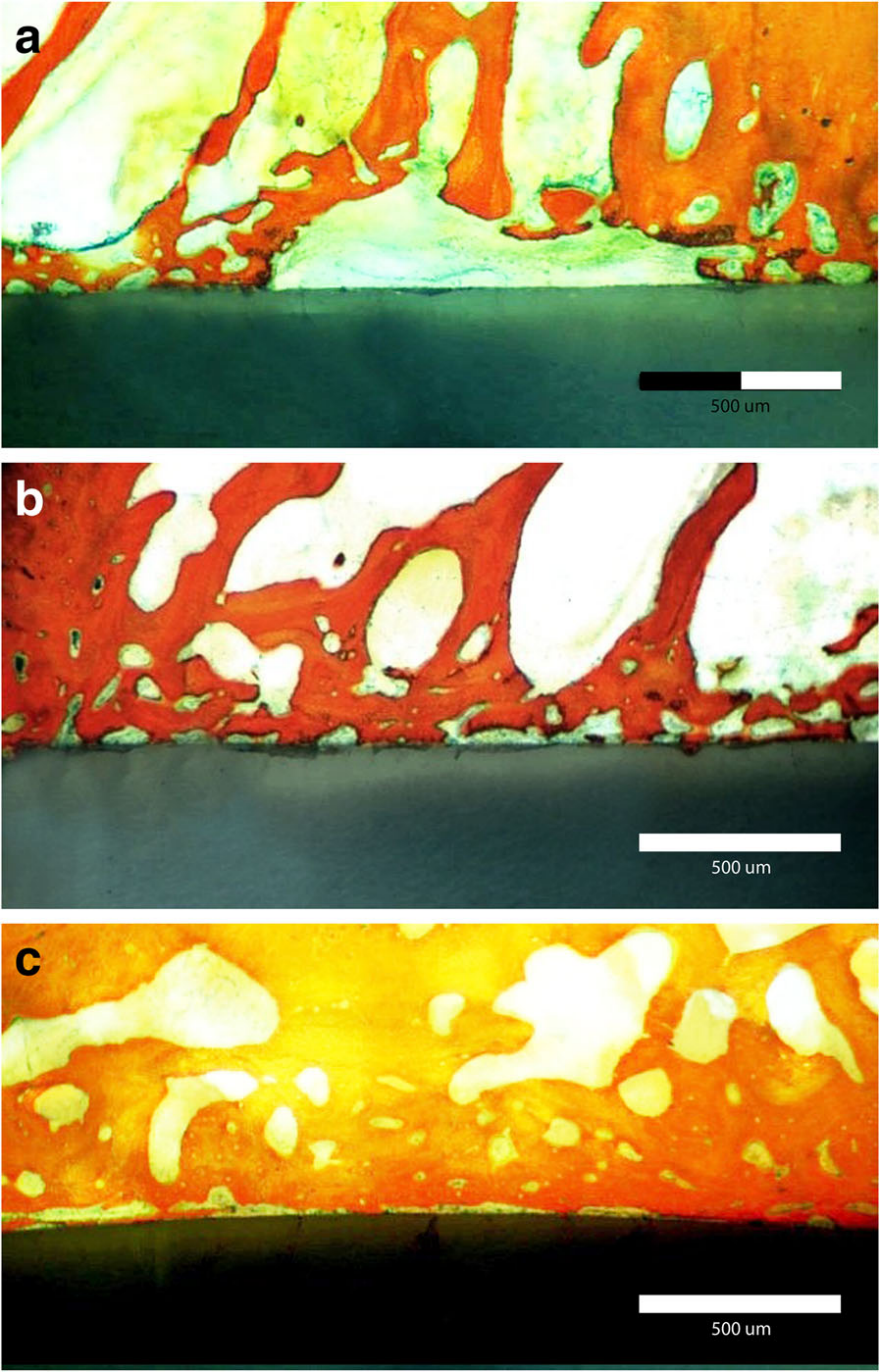
**a** Stained histomorphometric section demonstrating bone implant contact of uncoated zirconia implant. **b** Stained histomorphometric section demonstrating bone implant contact of HA–hybrid–zirconia surface. **c** Stained histomorphometric section demonstrating bone implant contact of PRP–hybrid–zirconia surface

Mercury porosimetry revealed comparable (*F* = 0.047, *P* < 0.9) average pore diameter (136.43 ± 2.76 nm) for all the prepared specimens in all groups (Fig. 1a). There was a significant reduction in total porosity percent (*F* = 848.960, *P* < 0.001) after application of coating materials: 9 ± 2% for HA–hybrid surface and 4 ± 1% for PRP-coated surface. EDX analysis of the extracted natural HA revealed that calcium phosphate ratio was 1.67 indicating successful extraction of pure hydroxyapatite (Fig. 1b). XRD pattern revealed the characteristic peaks specific for the hexagonal HA crystal phase represented by (211), (112), and (300 peaks), which proved the phase purity of the extracted HA. High-resolution XRD of uncoated specimen detected only one crystal phase, the tetragonal phase. HA peaks were detected on the hybrid-coated surface (Fig. 1c). EDX detected standard chemical composition of zirconia and HA particles. Atomic force microscopy revealed nano-porosity created as a result of selective infiltration etching surface treatments (Fig. 1d). SEM images of uncoated specimens revealed the presence of three-dimensional networks of nano-pores on the treated surface. Images of the hybrid–zirconia surfaces revealed the presence of agglomeration of HA nano-particles filling the porous surface (Fig. 2). Examination of histological sections indicated significantly higher (*F* = 14.6, *P* < 0.001) amount of newly formed bone (BIC%) around HA–bioactive–hybrid surfaces (79.8 ± 3%) and PRP-coated surfaces (71 ± 6%) compared to the uncoated surfaces (49 ± 8%) (Fig. 3).

## Discussion

Several techniques were previously tested for coating hydroxyl apatite particles in the surface of implants as the following: thermal (plasma) spraying [39], dipping coating [30], electrochemical deposition [40], sputter coating [41], pulsed laser deposition [42], and sol-gel technique [43]. Many parameters determined the performance of HA coating both in vitro and in vivo, including chemical composition, crystallinity and purity, surface morphology, porosity, and thickness. These parameters differ from one technique to the another not to mention the effect of varying the operating parameters of each technique [39]. In this study, HA particles were ground to the nano-scale (100 nm) to be incorporated into the three-dimensional nano-pores, with average diameter of 136.43 ± 2.76 nm, thus using the modified zirconia surface as a carrier to the bioactive particles either hydroxy apatite or platelet-rich plasma.

Immersion of a nano-porous zirconia implant in a solution of bioactive materials either HA or PRP created a unique bioactive–hybrid ceramic surface that enhanced the tissue interaction and healing mechanism around the inserted implants. The advantage of this hybrid ceramic surface is that the coated layer is adsorbed few microns beneath the surface, thus reducing any chance of delamination and debonding. Moreover, a nano-rough surface is known to enhance implant stability and facilitate improved implant–bone contact, which was directly observed in this study. The proposed hypothesis was accepted.

**Fig. 4:**
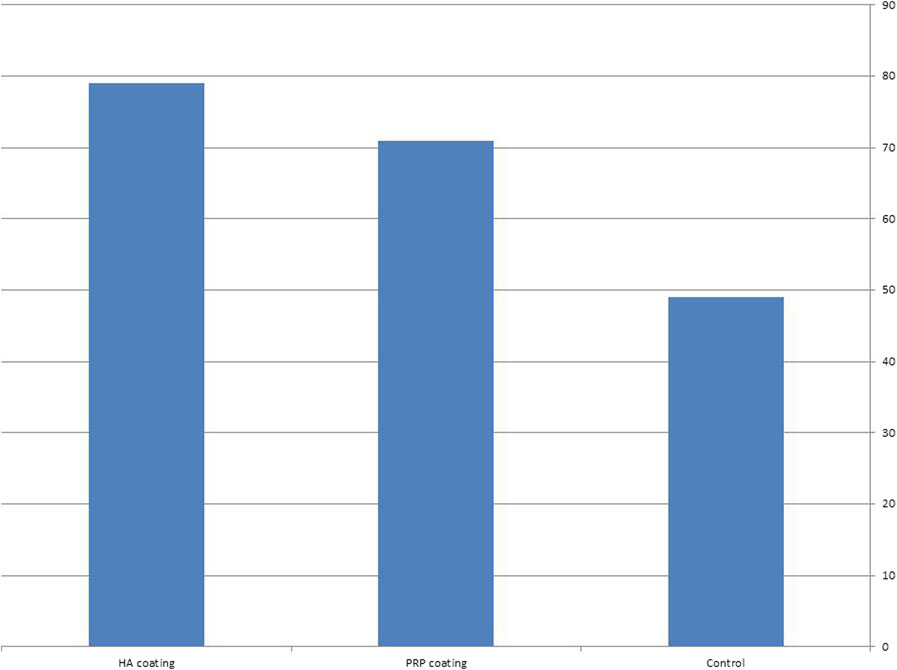
Bone implant contact of different test groups

Histomorphometric analysis are in accordance with many studies, which reported similar findings [44–48]. A histological study used partially stabilized zirconia (Y-TZP) coated with a thin carbonate-containing hydroxyapatite (CA) showed a significantly higher bone-to-implant contact ratio and more bone mass after insertion in the femoral trabecular bone of rabbits [49]. Also, in 2015, it was reported that pre-osteoblast cells exhibited higher proliferation rate on HA-coated zirconia than those grown on uncoated zirconia plants, while gene expression analysis indicated good osteogenic responses on HA-coated [50]. Similar successful findings were observed using PRP coating [51–57]. It was suggested that PRP alone could not induce new bone formation until 6 weeks after implantation, while PRP/HA composite activated osteogenic cells, resulting in enhanced bone formation [58] (Fig. 4).

On the other hand, although both the extracted natural HA and PRP bioactive coatings were autologous in nature, the hybrid–HA–zirconia implants showed a higher BIC% than the hybrid–PRP implants, which is directly related to the ability of bioactive HA to promote attachment and proliferation of matrix producing bone cells on its carbonated apatite surface, which has the same surface chemistry as natural bone [7, 8, 59]. Moreover, PRP has a limited time of effectiveness as it acts only in the early phase of clot formation and healing process while the effect of HA particles could be extended over several months.

It is worth mentioning that this significant enhancement in the peri-implant bone healing and osseointegration proves successful incorporation and absorption of the HA nano-particles and the PRP solutions into the nano-porous surfaces of the zirconia implants, thus creating a unique bioactive–hybrid surface, which contributes to enhancing the biological responses at the bone–implant interface and consequently optimizing the overall success rate of the coated implants. Being few microns thick, there is no fear of delamination or detachment observed with other coating techniques. Further studies are needed to optimize the design and performance of these hybrid surfaces.

## Conclusions

Within the limitations of this study, hybrid–zirconia surface enhanced osseointegration in rabbit model. The proposed hypothesis was accepted.
